# Iron Smelting

**Published:** 1881-01

**Authors:** 


### IRON SMELTING.

Iron ores contain iron and oxygen, combined with silica or alumina. If we deprive them of their oxygen, we get metallic iron. To do this Duryea feeds them into his iron or steel furnace, which is a 60 to 70 foot horizontal cupola, inclined and revolving, say, once in two minutes. Ores crushed to walnut size or smaller are fed into the upper end, mixed with an equal weight of coal dust or slack, and sufficient lime to make a liquid slag. As they descend, the carbon evolves $CO$ gas, which, taking up the oxygen of the ores by chemical preference, passes out of furnace. Now, then, if we have given them time enough in this gas to deprive them of all their oxygen, their reduction to metallic state is complete, and they are prepared for the next or melting stage. Here comes in the agency of the flux, as, if we add no alkali the iron and silica fuse, and results in a silicate of iron or black glass, but if lime (or preferably feldspar) be added, the silica and lime unite preferably, and the slag is lighter colored, while the metallic iron, in the shape of sponge iron, will be the result. To agglutinize this sponge iron a large metal bath of melted pig iron is necessary. This is done by throwing in at the beginning with the ores, pig-iron, which, melting, runs down into the basin or puddling hearth in the lower end of the cylinder. The sponge iron dropping into this bath, the slag separates and rises to the top, while the metallic iron, by gravity, settles to the bottom. Now revolve the cylinder till the tap-hole, which has been open, allows the slag to run off, and the iron, with its 5 per cent carbon, may be tapped off as pig iron in suitable sand trenches. Suppose we wish to make steel or wrought iron. After the slag is drawn off, the tap-hole is closed, the cylinder is set to revolving, feeding of ores withheld for half an hour. The oil nearly all shut off and oxygen of the air blast deprives the melted metal of its carbon, the mechanical puddling and cooking soon forms pasty blooms of wrought or puddled iron, the time required for this being from 15 to 30 minutes, while by hand-puddling, it is about two hours. As this mechanical movement does its work more effectually than is possible by handling with rod, the product should be more uniform. The blooms are now to be dumped out of the door into squeezers, which should be underneath the cylinder; after the squeezers have squeezed out the scoria or adhering slag they are ready for the hammer. If the ores contain phosphorus their dephosphorization by chlorine gas at any intense blow-pipe heat will be accomplished as follows: Chloride of sodium (salt) is fed in Hopper R. as the ores are melting. Chlorine, being set free by the decomposition of the salt uniting with phosphorus at about 3,500 F. heat, forms chloride of phosphorus, which is eliminated.

